# A transcriptomic approach to elucidate the physiological significance of human cytochrome P450 2S1 in bronchial epithelial cells

**DOI:** 10.1186/1471-2164-14-833

**Published:** 2013-11-26

**Authors:** Thushara W Madanayake, Ingrid E Lindquist, Nicholas P Devitt, Joann Mudge, Aaron M Rowland

**Affiliations:** 1 Department of Chemistry and Biochemistry, New Mexico State University, Las Cruces, NM 88003, USA; 2 National Center for Genomics Research, Santa Fe, NM 87505, USA

**Keywords:** CYP2S1, BEAS-2B, Retinoic acid, Arachidonic acid, RNA Seq, Orphan, shRNA, PGE2

## Abstract

**Background:**

Cytochrome P450 2S1 (CYP2S1) is an orphan P450 with an unknown biological function. Data from our laboratory and others suggest that CYP2S1 may have an important physiological role in modulating the synthesis and metabolism of bioactive lipids including prostaglandins and retinoids. CYP2S1 expression is elevated in multiple epithelial-derived cancers as well as in the chronic hyperproliferative disease psoriasis. Whether CYP2S1 expression in proliferative disease is protective, detrimental, or neutral to disease progression remains to be determined. Two human bronchial epithelial cells (BEAS-2B) were constructed to represent chronic depletion of CYP2S1 using short-hairpin RNA (shRNA) silencing directed toward the 3’UTR (759) and exon 3 (984) of the CYP2S1 gene and compared with a non-targeting shRNA control (SCRAM). Both CYP2S1 mRNA and protein were depleted by approximately 75% in stable cell lines derived from both targeted shRNA constructs (759 and 984). To elucidate the biological significance of CYP2S1, we analyzed transcriptome alterations in response to CYP2S1 depletion in human lung cells.

**Results:**

RNA-sequencing (RNA-seq) analysis was performed to compare the transcriptome of the control (SCRAM) and the CYP2S1-depleted (759) BEAS-2B cell lines. Transcriptomes of the replicates from the two cell lines were found to be distinct populations as determined using Principal Component Analysis and hierarchical clustering. Approximately 1000 genes were differentially expressed in response to CYP2S1 depletion. Consistent with our previous phenotypes, DAVID analysis revealed altered regulation in key pathways implicated in cell proliferation and migration. Transcriptomic profiles were also consistent with the metabolism of proposed endogenous substrates. Pathway analysis also revealed significant expression changes within mTOR signaling, a critical pathway in cell growth. To determine whether these changes manifest as altered cell size, cell diameter and volume were calculated, revealing that CYP2S1 depletion promotes cell growth in BEAS-2B cells.

**Conclusions:**

These data suggest that pathway analysis of sequence-based gene expression is a powerful method to identify pathways and phenotypic alterations in response to changes in orphan enzyme expression. Our results suggest a novel role for CYP2S1-mediated metabolism in modulating BEAS-2B cell size. These findings warrant further studies on CYP2S1 regulated pathways to elucidate potential substrates of CYP2S1.

## Background

Although the human genome was declared complete nearly a decade ago [1] and many of the proteins linked to these sequences have been identified, the challenge remains to characterize the functional significance of these proteins. It is particularly difficult to elucidate the metabolic activity of enzymes with no known substrate or critical function. Cytochrome P450s (CYPs) are heme-containing metabolic enzymes that typically catalyze the oxidation of endogenous and xenobiotic chemical substrates. Although these enzymes demonstrate a critical role in the metabolism of ~75% of all xenobiotic substrates, less than half of these enzymes have critical physiological functions [2-4]. A recent analysis of the human genome revealed approximately 57 distinct human CYPs [4]. Roughly one-quarter of these CYPs are classified as orphans with little or no knowledge of their substrates and physiological significance. One of the most recently identified CYPs, Cytochrome P4502S1 (CYP2S1), was identified through a bioinformatics approach [5] and is among these orphan P450s.

CYP2S1 is likely to play an important role in regulating endogenous metabolism. CYP2S1 expression is sensitive to regulation by endogenous and exogenous chemicals. Retinoic acid significantly elevated CYP2S1 at the mRNA and protein level in a variety of human epithelial cells [6], including human lung cells [7]. Oxygen deprivation within cultured cells also resulted in significant elevation of CYP2s1 mRNA within mouse hepatoma Hepa-1 cells [8]; however, a similar treatment in human monocytes did not alter expression [9]. CYP2S1 expression was elevated in response to agonists of the arylhydrocarbon receptor, AhR, including dioxin and 3-methylchloranthrene (3-MC) [10] and components of cigarette smoke [11]. Conversely, anti-inflammatory glucocorticoid agonist treatment of human lung cells at physiologically relevant concentrations significantly depleted CYP2S1 mRNA through epigenetic modulation of CYP2S1 [12]. CYP2S1 was also elevated in chronic hyperproliferative diseases, including psoriasis [6] as well as multiple epithelial cancers [13-16]. It is likely that both transient and chronic changes in CYP2S1 expression alter metabolic activation of potential endogenous substrates and may reveal an important physiological role for CYP2S1.

Although it has been shown to effectively metabolize cancer therapeutics of the anthraquinone (AQ4N) [17,18] and benzothiazole family (GW610 and SF203) [19], CYP2S1 is still considered an orphan with no known endogenous substrates. Candidate substrates have been identified and include the bioactive lipids all-trans retinoic acid (RA) [6,20,21] and metabolites of the arachidonic acid inflammatory cascade (in particular metabolites of the cyclooxygenase (prostaglandins) and lipoxygenase (HETES) pathways [9,22]. CYP2S1-mediated metabolism of these lipids appears to require atypical metabolism (peroxide shunt pathway) and whether CYP2S1 contributes to the metabolism of these bioactive lipids is controversial [18]. However, *in vitro* cellular assays in human and rodent cells appear to be consistent with metabolism of these endogenous substrates [9,22,23]. Previous work in our laboratory, examined the impact of CYP2S1 depletion on human bronchial epithelial (BEAS-2B) cells [23]. Depletion of CYP2S1 in these cells led to enhanced cell proliferation and migration. Cell proliferation was, in part, attributed to modulation of arachidonic acid cascade, resulting in elevated levels of the inflammatory prostaglandin (PGE2) [23]. The etiology of the change in migration, however, is still unclear.

Elucidating the physiological significance of an orphan cytochrome P450s is a complicated process hampered by difficulties in isolation and purification of CYPs as well as identifying possible substrates from a myriad of potential chemicals. Historically, a trial-and-error approach has been used to identify possible substrates. However, this approach is time and resource intensive and neglects chemicals outside of the chemical library. Recently, the Guengerich lab has successfully utilized advances in methodology and mass spectrometry analysis to identify endogenous substrates for a number of cytochrome P450s, including orphans [24,25]. This approach is a significant advance in identification of novel endogenous substrates. However, it does not directly demonstrate the physiological impact of changes in P450 expression within human cells. To elucidate the physiological significance of alterations in P450 expression on biological pathways, we utilize next generation sequencing as a novel, unbiased approach to identify transcriptional changes in biological pathways within human cells. Specifically, we compare the transcriptomic profiles of two human bronchial epithelial (BEAS-2B) cell lines with differential CYP2S1 expression: CYP2S1 depleted (759) vs. control (SCRAM). Previous work in our lab demonstrated that CYP2S1 depleted cells enhanced cell proliferation and migration [23]. We illustrate how pathway analysis of sequence-based differential expression results identified the molecular pathways perturbed in response to CYP2S1 depletion, provided insight into unexpected modes of action, and informed follow up experiments. Here we report how transcriptomic profiles are consistent with published phenotypes and proposed endogenous metabolism. Additionally, our results reveal novel changes in the mTOR signaling pathway, which has been linked to cell size [26]. We pursued this phenotype experimentally and confirmed a significant increase in cellular diameter and volume in CYP2S1 depleted cells, suggesting a previously unknown role for CYP2S1-mediated metabolism in the regulation of cell growth.

## Results and discussion

### RNA-sequencing Analysis of CYP2S1 depleted human bronchial epithelial cells (BEAS-2B)

**Figure 1:**
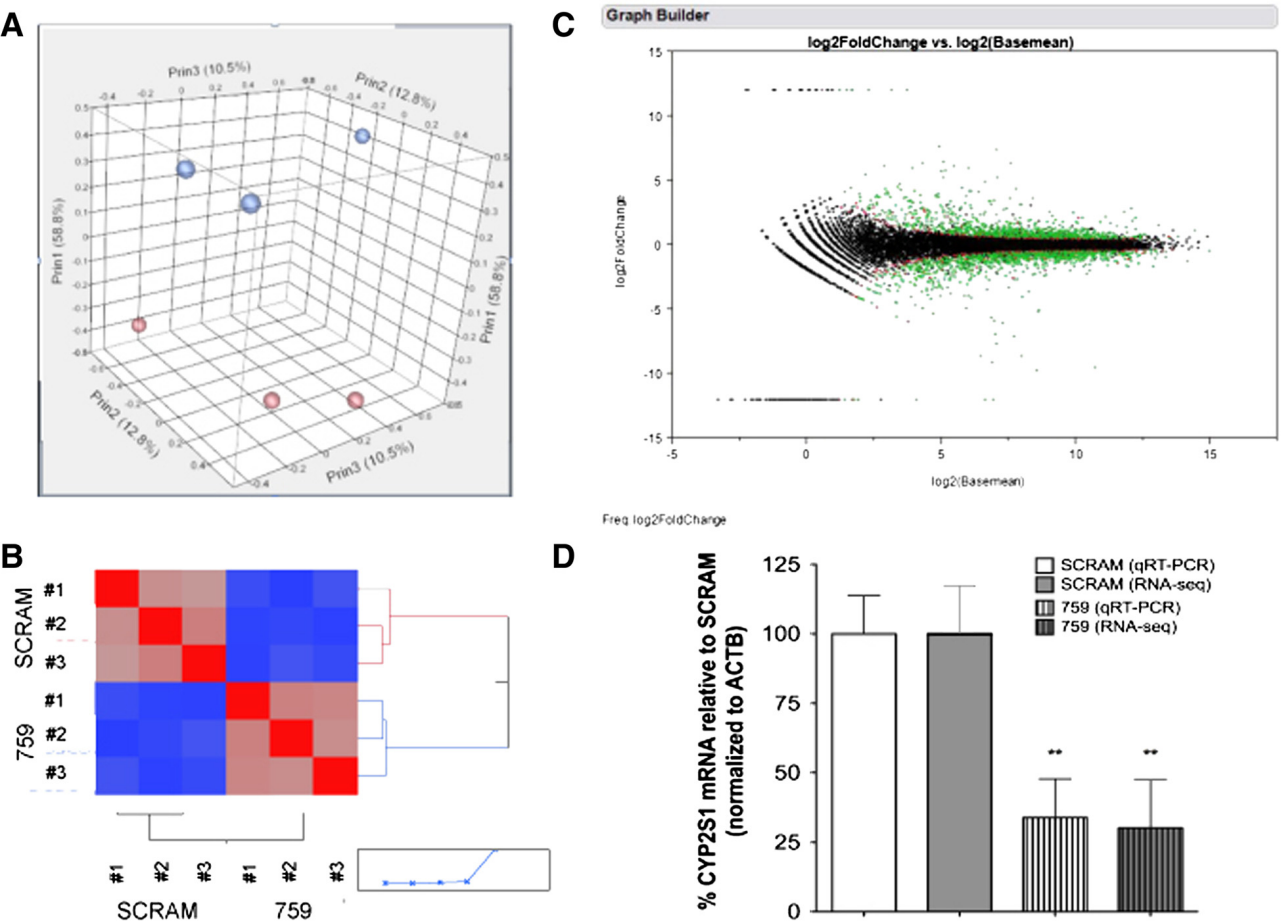
**RNA sequencing data quality and verification of CYP2S1 depletion in human bronchial epithelial cells. A**. Principal component analysis shows significant differences between biological samples with normal (SCRAM, red) and reduced (759, blue) expression of CYP2S1. **B**. Heat map analysis reveals similarities between the biological replicates within each condition. **C** The spots on the MA plot represent each gene within the analysis. The x-axis displays the log2 basal expression for each gene, while the y-axis represents the log2 change in expression relative to (SCRAM) control. FDR adjusted significant changes (p < 0.05) are represented as green dots. **D**. Comparison of CYP2S1 expression normalized to ACTB using either RNA-seq (grey) or qPCR (white). ** represents significant differences (p < 0.01) between CYP2S1 expression in SCRAM controls (no fill) and CYP2S1 depleted (vertical lines) cells.

To determine whether alterations in the transcriptomic profiles would reveal the functional significance of CYP2S1 in human bronchial epithelial (BEAS-2B) cells, we performed short hairpin RNA (shRNA) silencing using sequences targeting the 3’UTR (759) and exon3 (984) to deplete CYP2S1 expression in BEAS-2B cells. The depletion of CYP2S1 mRNA and protein was confirmed in comparison to non-targeted shRNA control (SCRAM) [23]. The clones exhibiting the greatest difference in CYP2S1 mRNA and protein expression (i.e. 759#7 and SCRAM#1) were further analyzed using RNA-sequencing. Total mRNA was isolated from three independent passages of each of CYP2S1 depleted (759) samples and scrambled controls (SCRAM) and prepared into 6 sequencing libraries. Samples were sequenced on the Illumina HiSeq 2000 platform, yielding an average of 11,824,726 1×50 nt short reads per sample. These filtered reads were aligned with GSNAP [27] to the human reference genome (GRCh37), binned by annotated gene coordinate, and uniquely aligning reads were summated to generate read count-based gene expression estimates. Principal Component Analysis and hierarchical clustering as implemented in JMP Genomics {6.0} were used to qualitatively assess transcriptome-wide similarities among biological replicates and between genotypes (759 vs. SCRAM). Principle Component Analysis reveals that the three biological replicates from each genotype tightly cluster with one another and the largest aspect of captured transcriptome variability separates samples based on genotype. This low variability within genotype increases our ability to find quantitative differences in gene expression that are truly attributable to differences in CYP2S1 expression (Figure 1-A,B).

To identify specific differentially regulated genes between CYP2S1 depleted (759) and scrambled control (SCRAM) in human bronchial epithelial cells, we used the negative binomial test of significance as implemented in the Bioconductor package DESeq with significance defined as an adjusted p-value ≤0.05 [27] (Figure 1C). CYP2S1 depletion resulted in the increased expression of 1159 genes while significantly reducing expression of 1326 genes. RNA-seq confirmed CYP2S1 depletion of approximately 3-fold in these samples. Quantitative PCR of CYP2S1 was performed (primers used as previously described [23] on each of the three biological replicates, and expressed relative to one of the most stable housekeeping genes identified in our study, b-actin (ACTB). The results demonstrate a high concordance in quantification of relative CYP2S1 expression between the two experimental methods (Figure 1D). A comprehensive list of differentially expressed genes is included in an additional file (Additional file 1).

### DAVID analysis reveals biological pathways associated with previously published phenotypes

**Figure 2:**
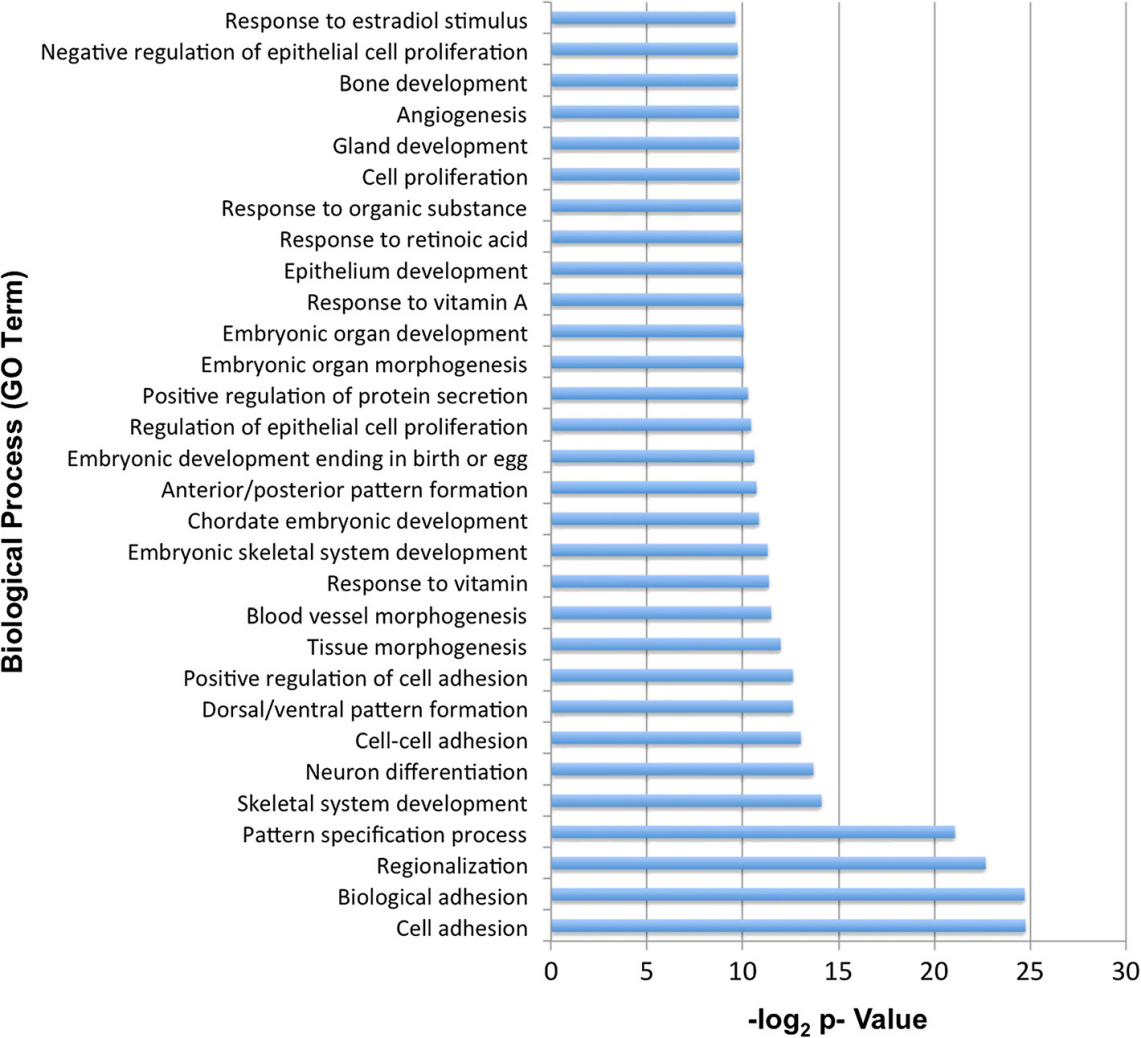
**Top GO BP terms in CYP2S1 depleted human bronchial epithelial cells.** The y-axis represents the top 30 biological processes and the x-axis displays negative log2 P values. Analysis was restricted to 995 genes, representing p < 0.01 and at least 2-fold change in expression.

To identify biological pathways regulated in response to CYP2S1 depletion in BEAS-2B, we performed pathway analysis using the Database for Annotation, Visualization, and Integrated Discovery (DAVID; http://david.abcc.ncifcrf.gov) [28,29]. DAVID analysis was performed on differentially expressed genes, identified using DESeq analysis (p < 0.01), with the additional criteria that the differences exhibit at least a 2-fold change in expression. This further restriction reduced the number of genes to 998 (430 up and 568 down). Functional annotations were available for 919 of these genes. The annotations were listed in terms of Kyoto Encyclopedia of Genes and Genomes (KEGG) pathways as well as Gene Ontology (GO) terms. Differentially expressed genes (p < 0.01) were used for KEGG pathway analysis and revealed 17 statistically significantly enriched KEGG terms (Table 1). The most highly significant KEGG terms [Modified Fisher Exact P value (EASE score) < 0.01] include cell cycle, lysosome, apoptosis, and prostate cancer. Other KEGG terms with a significant number (EASE score ≤0.05) of genes altered in response to CYP2S1 depletion include sphingolipid metabolism, TGFβ and mTOR signaling pathways. DAVID enrichment analysis was also performed with GO biological process (BP) terms. Complete GO BP term annotations are included in supplementary information (Additional file 2). The top 30 GO BP terms are shown in Figure 2. The top GO annotations included terms related to cell adhesion (cell adhesion, biological adhesion, cell-cell adhesion), cell proliferation (negative regulation of epithelial cell proliferation, cell proliferation, regulation of epithelial cell proliferation), and retinoic acid regulation (response to vitamin, response to vitamin A, response to retinoic acid). The identification of genes involved in cell proliferation and adhesion are consistent with enhanced cell viability and migration phenotypes identified previously in these CYP2S1 depleted (759 and 984) BEAS-2B cells [23].

**Table 1:** Statistically significant KEGG classifications of differentially expressed genes in CYP2S1 depleted cells

**KEGG term**	**Count**	%*	**P value (EASE score)**	**Fold enrichment**
Cell Cycle	32	1.18	7.96E-04	1.83
Lysosome	29	1.07	2.49E-03	1.77
Apoptosis	22	0.81	7.43E-03	1.80
Prostate cancer	22	0.81	9.73E-03	1.76
Sphingolipid metabolism	12	0.44	1.52E-02	2.19
TGF-beta signaling pathway	21	0.78	1.53E-02	1.72
Nucleotide excision repair	12	0.44	3.62E-02	1.95
Pancreatic cancer	17	0.63	3.77E-02	1.68
N-Glycan biosynthesis	12	0.44	4.86E-02	1.86
mTOR signaling pathway	13	0.48	5.17E-02	1.78
Aminoacyl-tRNA biosynthesis	11	0.41	5.20E-02	1.91
Glycine serine and threonine metabolism	9	0.33	5.85E-02	2.07
Bladder cancer	11	0.41	6.01E-02	1.87
Pathways in cancer	57	2.11	6.03E-02	1.24
Glutathione metabolism	12	0.44	8.14E-02	1.71
Nitrogen metabolism	7	0.26	9.14E-02	2.17
Non-homologous end-joining	5	0.19	9.65E-02	2.74

*Indicates the percentage of genes in each pathway from 2702 genes mapped to KEGG.

### CYP2S1 depletion evokes changes in P450s that metabolize bioactive lipids

CYP2S1 does not appear to be required for survival, since the CYP2a (4/5) bgs-null mouse (which includes a CYP2s1 knockout) is both viable and fertile [30]. It is likely that there is redundancy between CYP2S1 and other P450s in endogenous metabolic substrates. Therefore the effect of CYP2S1 depletion on CYP expression was determined. A total of 57 CYPs were analyzed in this RNA-seq experiment. The majority of CYPs (86%) were equally divided among CYPs that were either not expressed (51%; base mean value <1.5 read) or were not significantly altered in response to CYP2S1 depletion (35%; p > 0.05). A total of 8 CYPs (14%) were significantly altered in response to CYP2S1 (759) depletion. Five of the eight exhibit at least a 2-fold increase (CYP4F11, CYP2J2, CYP4Z1) or decrease (CYP2S1, CYP1A1) in expression compared to scrambled (SCRAM) control (Table 2). Two of these enzymes (CYP4F11 and CYP4Z1) are still considered orphans [2-4], although hydroxylation of bioactive lipids has been demonstrated in recent studies [31,32]. Interestingly, each enzyme altered in response to CYP2S1 depletion are involved in metabolism of bioactive lipids including arachidonic acid (Table 2).

**Table 2:** Cytochrome P450s altered in CYP2S1 depleted cells

**Altered in CYP2S1 depleted BEAS-2B**	**Fold change & p value**	**Substrate status**	**Evidence for endogenous substrate metabolism**
CYP2S1	-3.2; p < 0.0001	Orphan	Retinoic acid [6,21,22]; prostaglandin metabolism [9,23]
CYP1A1	-3.6; p < 0.05	Xenobiotic	Estrogen, bilirubin, melatonin, arachidonic acid. Reviewed in [33]
CYP4Z1	4.2; p ≤ 0.05	Orphan	Saturated and unsaturated fatty acid hydroxylation including lauric acid (C12:0, myristic acid (C14:0); 11,14-eicosadienoic acid (C20:2); (C20:4) [31]
CYP4F11	2.7; p ≤ 0.001	Orphan	Fatty Acids (w-2,-3, -4, -5) [25,32]
CYP2J2	3.6; p < 0.05	Fatty Acid	Arachidonic acid epoxidation to epoxyeicosatrienoic acids (EETS). Reviewed in [34]

### Pathway analysis for the synthesis and metabolism of proposed lipids substrates

CYP2S1-mediated metabolism of endogenous substrates remains controversial. However, there exists published biochemical and cellular evidence to suggest CYP2S1 mediated metabolism of the bioactive lipids including all-*trans* retinoic acid [6,20,21] as well as metabolic products of the arachidonic acid metabolism [9,22,23]. Transcriptional responses were evaluated for each pathway to determine whether physiological responses to CYP2S1 depletion were consistent with its proposed role in lipid metabolism.

### Transcriptome analysis of retinol metabolism in CYP2S1 depleted cells

All-*trans* retinoic acid (RA) is transported to the cell as retinol (Vitamin A) and converted to retinal via oxidoreductases (ADH and SDR). Retinal is subsequently bioactivated to RA via retinal dehydrogenases (RALDHs) [35]. Once formed, RA is inactivated via P450 oxidation to a variety of metabolites. The CYP26 family is the most effective at oxidizing RA [36-39]. Other CYPs’ (CYP1A, CYP2ABC, CYP3A, CYP4A) oxidation and conjugation of retinoic acid to multiple hydroxylated products as well as the glucuronide conjugate via UDP glucuronosyltransferase (UGT2B7) [40], respectively, is believed to inactivate RA. Heterologous expression of CYP2S1 has yielded contradictory results that either demonstrate [6,20] or fail to show [17,18] CYP2S1-mediated metabolism of RA. However, CYP2S1 expression within a cellular context in both chinese hamster ovary (CHO) cells and human keratinocytes (HaCaT) cells demonstrate that CYP2S1 contributes to retinoic acid metabolism in cells [21]. To function, RA is shuttled to either cellular retinoic acid binding protein (CRABPII) or fatty acid binding proteins (FABP5) [41], depending on their expression levels within the cell. Once bound, RA is delivered by CRABP and FABP5 to retinoic acid receptor (RAR) or peroxisome proliferating receptor (PPAR), respectively, where it alters transcription of numerous downstream targets.

**Figure 3:**
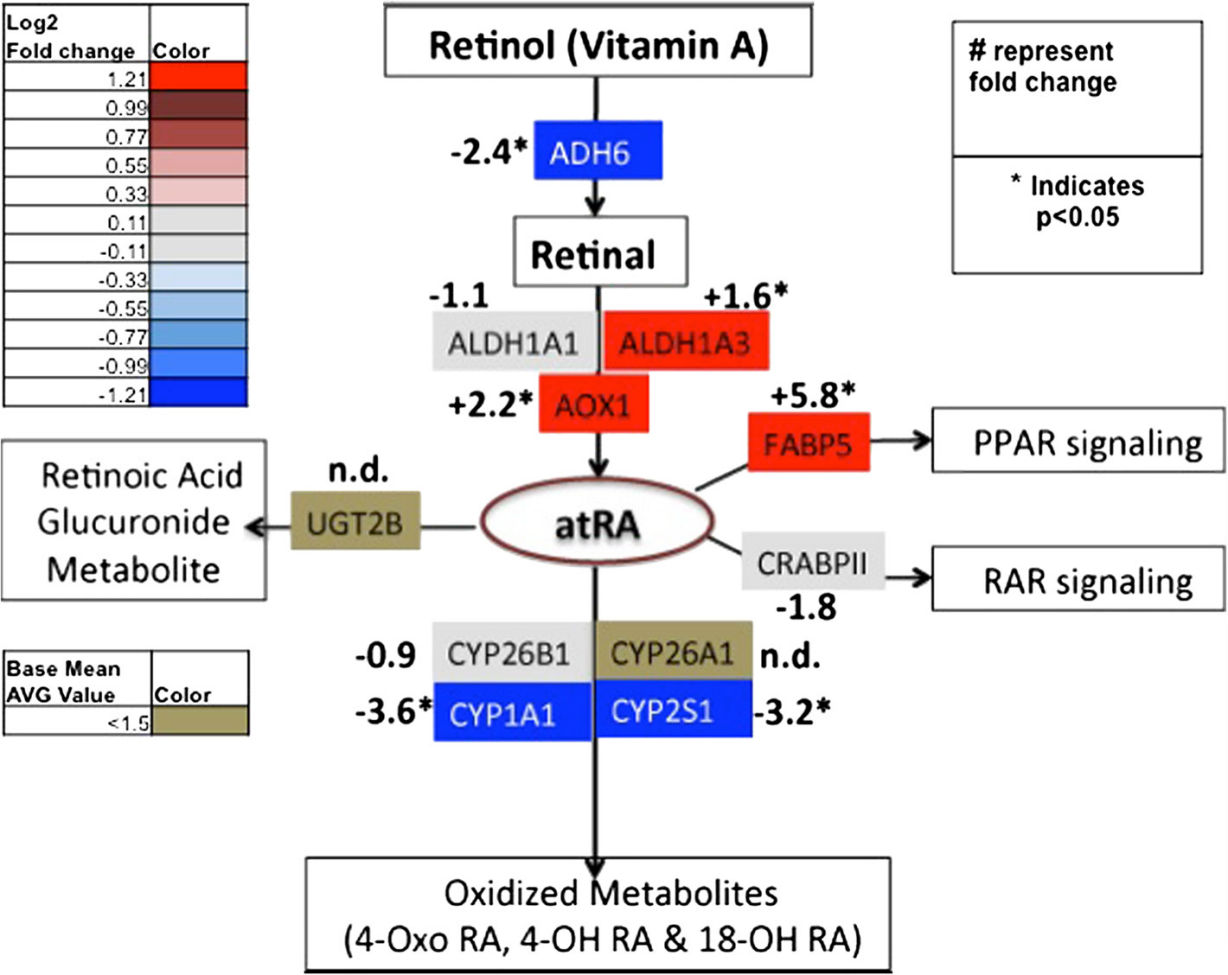
**The effects of CYP2S1 depletion on retinoic acid metabolism.** A simplified version of genes involved in retinol metabolism, focusing specifically on RA synthesis from retinol and retinal, RA metabolism through P450 oxidation and glucuronide conjugation, and RA signaling through either PPAR or RAR mediated pathways. Grey indicates no significant change in expression. Gold represents very low expression (i.e. base mean average value is <1.5). Shades of red and blue indicate a significant (p < 0.05) increase and decrease in expression in CYP2S1 depleted cells, respectively. Numerical values represent fold change in RNA-seq. * indicates the significant (p < 0.05).

According to the RNA-seq data, CYP2S1 depletion clearly resulted in significant alterations in the transcriptome of genes involved in retinoic acid metabolism. Response to vitamin A and response to retinoic acid metabolism were identified as key GO terms (Figure 2). CYP2S1 depletion in BEAS-2B cells resulted in an overall down regulation of the RA metabolism compared to scrambled control (Additional file 3). Many of the key enzymes involved in metabolism of retinol were not expressed in either CYP2S1 containing (SCRAM) or CYP2S1 depleted BEAS-2B cells, including CYP26A1, RPE65, and UGT2B7. CYP26A1 is the most efficient metabolizing cytochrome P450 at oxidizing retinoic acid, with affinity in the low nanomolar range [36]. CYP26B1 is the only CYP26 family expressed in BEAS-2B cells and has demonstrated similar catalytic activity toward RA [37-39]. RNA-seq data failed to demonstrate altered CYP26B1 expression in response to CYP2S1 depletion. Interestingly, upon closer inspection (Figure 3) we found that other enzymes involved in converting retinal to RA (i.e. AOX1) were significantly upregulated in CYP2S1 depleted cells, suggesting a metabolic flux possibly resulting in elevated RA (Figure 3). Additionally, the largest increase in expression was observed in FABP5 (5.8 fold increase) suggesting that the retinoic acid flux may be diverted into the PPAR signaling rather than metabolic inactivation. However, experimental validation is required to test these hypotheses.

### Transcriptome analysis of arachidonic acid metabolism in CYP2S1 depleted cells

CYP2S1 has been shown to metabolize products of the two major metabolic pathways of the arachidonic acid (AA) cascade, lipoxygenase and cyclooxygenase, in the presence of lipid peroxides [22]. Fromel et al also demonstrated CYP2S1-mediated epoxidation of a variety of bioactive lipids including arachidonic acid to epoxyeicosatrienoic acid (EETs) in sf-9 cells expressing CYP2S1 [9]. CYP2S1 depleted human BEAS-2B [23] and monocyte-derived macrophages [9] show increased levels of the inflammatory prostaglandin, PGE2. Conversely, over-expressing human CYP2S1 in rodent cells reduced synthesis of products of the cyclooxygenase pathway, PGE2 and PGD2 [22]. Based on published data, as well changes in P450 expression (Table 2), we would have anticipated significant changes in arachidonic acid metabolism in CYP2S1 depleted cells (759) compared to controls (SCRAM). However, arachidonic acid metabolism was not identified in the KEGG or GO terms. In fact, the arachidonic acid GO term did not appear in our most stringent (p < 0.01) criteria, but it did show up as #175 when the p-value was relaxed (p < 0.05) (Additional file 4). It is possible that the inability to detect transcriptional changes within this pathway could suggest that either: i) subtle transcriptional changes, undetected as significantly different, in this pathway are sufficient to elicit significant changes in AA metabolism, or ii) pathway analysis relies on *a priori* knowledge of AA metabolism, which may not be sufficient to clearly discern this pathway. These data suggest that transcriptome analysis may underrepresent potentially important pathways altered in response to changes in CYP2S1 expression.

**Figure 4:**
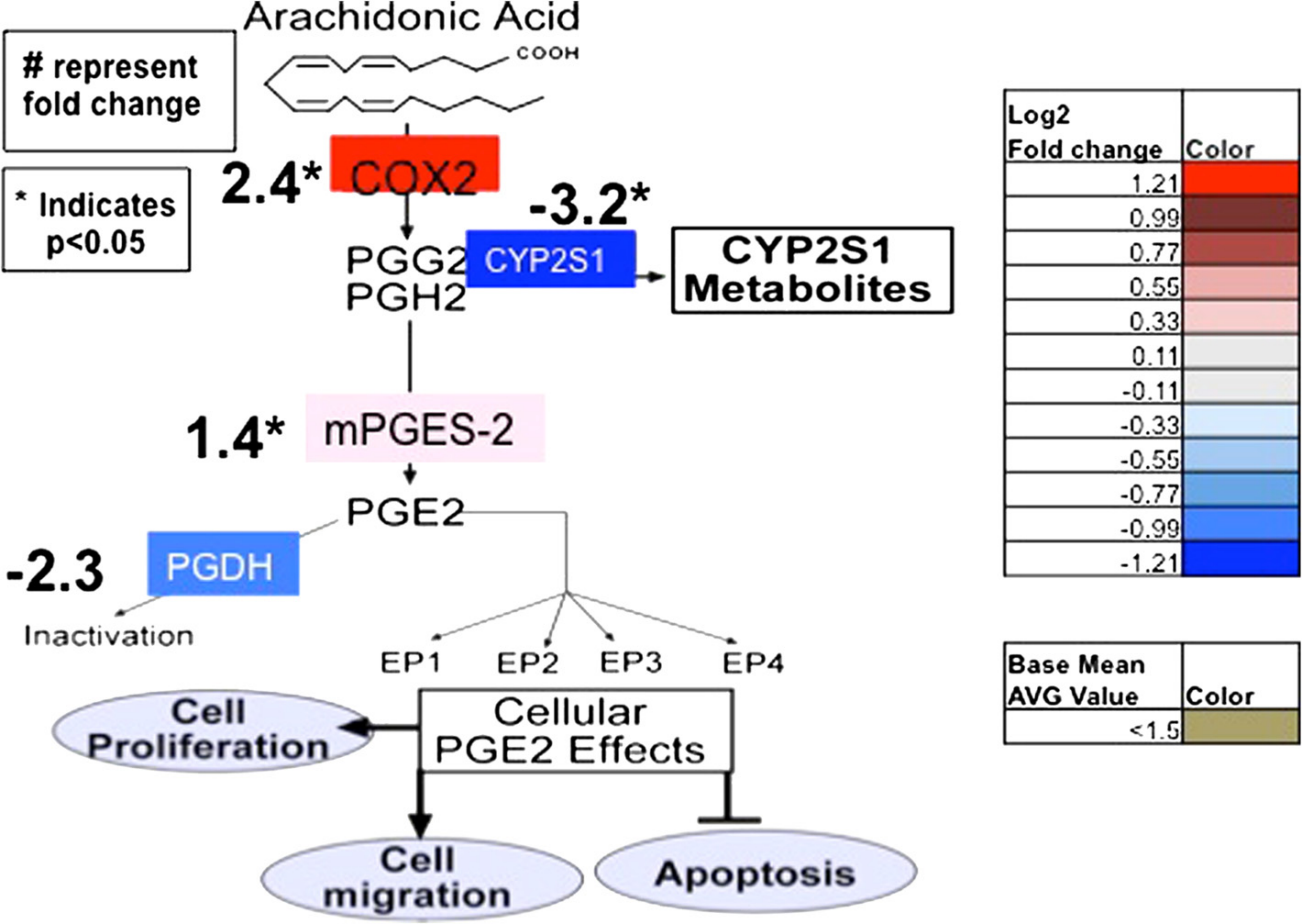
**The effects of CYP2S1 depletion on arachidonic acid metabolism.** A simplified version of the cyclooxygenase pathway within the arachidonic acid metabolism pathway, focused on published cellular modulation of prostaglandin synthesis. Grey indicates no significant change in expression. Gold represents very low expression (i.e. base mean average value is <1.5). Shades of red and blue indicate a significant (p < 0.05) increase and decrease in expression in CYP2S1 depleted cells, respectively. Numerical values represent fold change in RNA-seq. * indicates the significant (p <0.05).

To determine the extent of regulation within the AA pathway, we examined genes within the KEGG AA pathway (Additional file 5). The main regulatory enzymes in prostaglandin production (i.e. cyclooxygenase 2 (PTGS2)), EET production (i.e. CYP2J2), and HETE production (i.e. CYP4F11) are all significantly upregulated. In contrast, key regulatory enzymes involved in the lipoxygenase pathway (i.e. LOX) were not significantly altered. Closer examination of the cyclooxygenase pathway (Figure 4) reveals modulation consistent with an increase in inflammatory prostaglandin, PGE2 production. Specifically, the enzyme converting AA to PGG2 and PGH2 [COX-2 (PTGS2)] is upregulated 2.4 fold. The prostaglandin synthase (PGES2) is also slightly but significantly elevated (1.4 fold). In contrast, the PGE2 degrading enzyme, 15-PGDH, is reduced by 2-fold. Transcriptional alterations within the cyclooxygenase pathway are consistent with elevated PGE2 levels identified previously [23]. Therefore, it is possible that analysis of subsets from metabolic pathways may be required to identify significant regulation.

### RNA-sequencing analysis reveals a novel phenotype in CYP2S1 depleted cells: differential regulation of cell size

To determine whether RNA-sequencing analysis could reveal novel functions or phenotypes associated with an orphan P450, we examined the literature for connections between the top KEGG pathways. Three of the top regulated pathways are involved in regulation of cell cycle, growth factor signaling, and mTOR signaling. A common connection between each of these is the regulation of cell size. In order to grow and divide, cells must double their cellular contents (Reviewed in [42]). CYP2S1 depleted BEAS-2B cells exhibit increased cell proliferation [23]. Cell cycle control was listed as the most significant change in the KEGG classification. The growth factor TGFβ, exhibits crosstalk with the mTOR pathway [43]. mTOR pathway is recognized as a central regulator in cell growth [26], and represents a potentially novel CYP2S1-regulated pathway.

**Figure 5:**
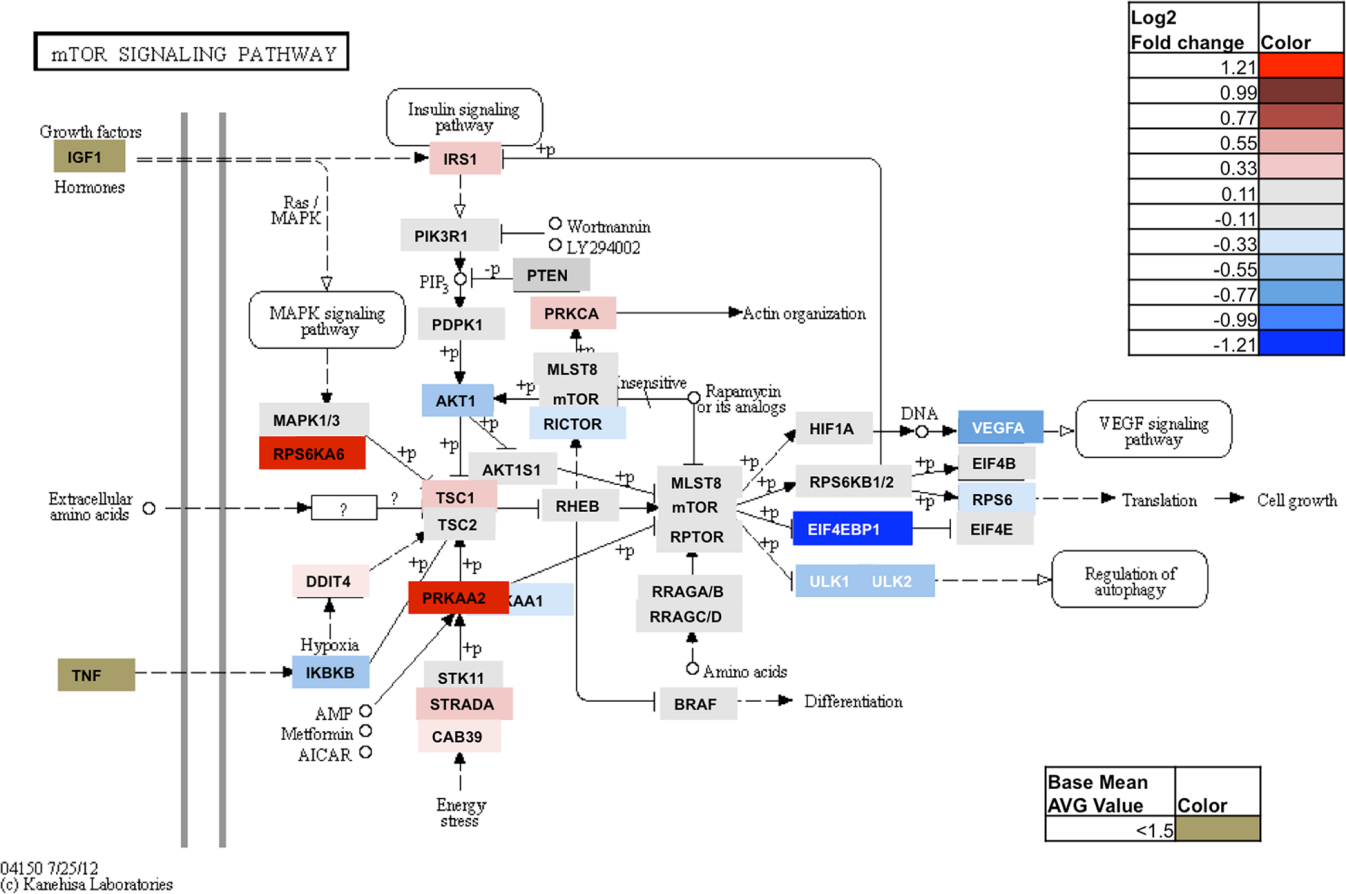
**The effects of CYP2S1 depletion on mTOR signaling.** Heat map data transposed onto the KEGG mTOR signaling pathway. Grey indicates no significant change in expression. Gold represents very low expression (i.e. base mean average value is <1.5). Shades of red and blue indicate a significant (p < 0.05) increase and decrease in expression in CYP2S1 depleted cells, respectively. Numerical values represent fold change in RNA-seq. * indicates the significant (p <0.05).

To probe which genes within the mTOR pathway were altered in response to chronic CYP2S1 depletion, RNA seq results for each gene in the mTOR signaling pathway (according to the KEGG and SABioscience array) were identified (Figure 5). A total of 24 out of 84 total genes (29%), identified by the mTOR PCR array as key regulated genes within the mTOR pathway, were identified through RNA sequencing as significantly altered (p-adjusted < 0.05) between CYP2S1 depleted (759) and scrambled controls (SCRAM). Regulation was similarly divided between the elevated (9 genes accounting for 10% of the genes) and reduced expression (15 genes accounting for 18% of the total). A total of 8 genes out of the 84 (9.5%) genes exhibit at least a 2-fold change in expression when compared to ACTB: Downregulated (5 genes: AKT1, EIF4EBP1, INSR, VEGFA, VEGFB); Upregulated (3 genes: DEPTOR, PRKAA2, and SGK1).

**Figure 6:**
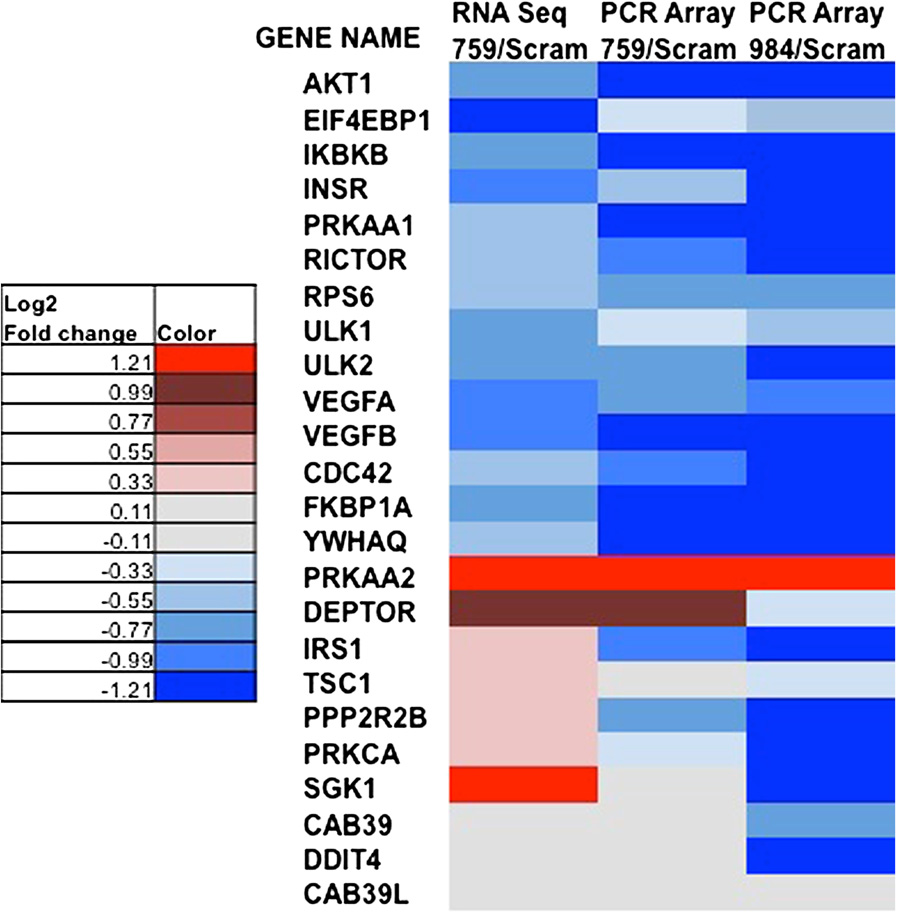
**Concordance between RNA seq and quantitative PCR array within genes of the mTOR signaling pathway.** A subset of 84 genes from the mTOR PCR array is compared between shRNA targets (759 and 984) and methodologies (i.e. RNA-seq and PCR array) using a heat map. Grey indicates no significant change in expression. Gold represents very low expression (i.e. base mean average value is <1.5). Shades of red and blue indicate a significant (p < 0.05) increase and decrease in expression in CYP2S1 depleted cells, respectively. All colored genes except grey indicate significant increases in RNA seq, p < 0.05.

mTOR PCR array (*SABioscience, Qiagen)* was used to validate transcriptional changes discovered by RNA-seq. PCR array results were used to establish concordance between approaches (using 759) and shRNA target (759 vs 984). A total of 84 regulated genes within the mTOR pathway and five different housekeeping genes (B2M, ACTB, GAPDH, HPRT1, and RPL13A) were quantified. In order to compare results between RNA-seq and PCR array, we identified ACTB as one housekeeping gene for which expression was consistent between CYP2S1 depleted cell lines (759 and 984) and scrambled controls (SCRAM). Thus both RNA-seq and quantitative PCR arrays were normalized to ACTB for comparison. The 24 genes exhibiting significant regulation are visualized using a heat map (Figure 6). Among the 24 genes that were significantly regulated, 19 out of 24 genes (80%) were similarly regulated using the PCR array on CYP2S1 depleted (759); whereas, 17 out of 24 (71%) were similarly regulated among CYP2S1 depleted (984). All of the 15 downregulated genes were confirmed using PCR array in both 759 and 984 (Figure 6). Interestingly, the upregulated genes did not demonstrate this concordance. Only 4 and 2 genes out of 9 were similarly regulated in the 759 and 984, respectively. Interestingly, only PRKAA2 demonstrated significant elevated expression between the two shRNA targets. Overall, our data demonstrate a good concordance between RNA seq and validation with qRT PCR arrays.

**Figure 7:**
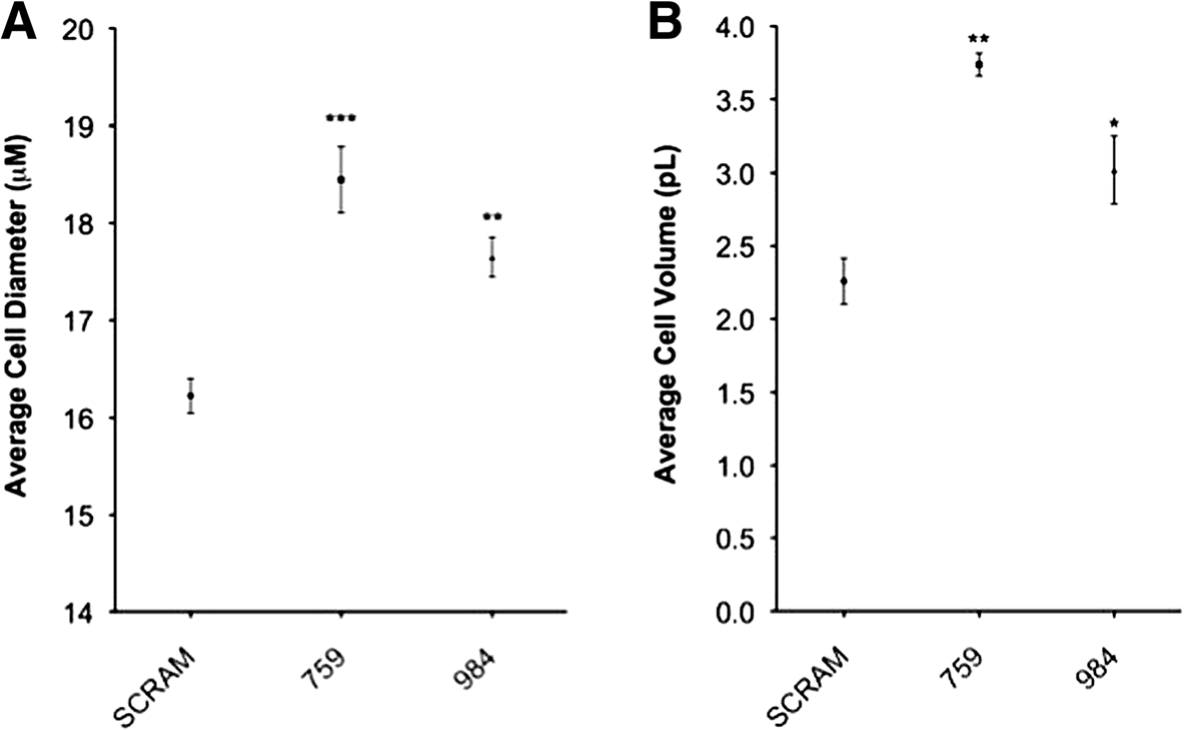
**Quantification and comparison of BEAS-2B cell diameter and volume. A**. Average cell diameter (μM) and **B**. volume (pL) in CYP2S1 depleted cells (759 and 984) vs. scrambled (SCRAM) control. ***, **, and * represent p values p < 0.001, p < 0.01, and p < 0.05, respectively.

Based on validated differences within the mTOR pathway and significant changes in cell cycle and TGFβ signaling, alterations in cell size were assessed. To determine changes in cell diameter and volume, we utilized the Millipore Scepter. Cells were cultured to approximately 80% confluence, trypsinized and counted. The average cell diameter and volume of the CYP2S1 depleted cell lines (759 and 984) and scrambled control (SCRAM) were graphed (Figure 7). Interestingly CYP2S1 depleted cells exhibit a significant ~13% increase in diameter (2 μM) and ~50% increase in volume (2pL). This increase cannot be attributed to subclonal variation, since two additional CYP2S1 depleted clones exhibit similar increases in cell size when compared to two distinct SCRAM controls (data not shown).

Only one gene, eukaryotic initiation factor 4E binding protein 1 (eIF4EBP1; log2 fold = -1.29, p < 0.001) met the criteria of both statistical significance p < 0.05 and 2-fold decrease in expression. eIF4EBP1 interacts with the translation initation factor eIF4E inhibiting the assembly of translation complex [44,45] The ratio of eIF4E/4E-BP is important for controlling translation of eIF4E-sensitive mRNA [46]. In this experiment, eIF4EBP1’s significant downregulation would be consistent with an increase in cell size in CYP2S1 depleted cells, since increased protein synthesis is required for cell growth.

We propose that elevated PGE2 observed in response to CYP2S1-mediated modulation of the cyclooxygenase pathway [23], may stimulate the mTOR pathway to promote cell growth. Recently, PGE2 was shown to promote phosphorylation and activation of Akt through the EP4 receptor in prostate cancer cells [47]. Furthermore, mitogen-stimulated activation of PI3K and Akt have been linked to the activation of the mammalian target of rapamycin complex I (mTORC1) [48]. mTORC1 kinase activity, in turn, stimulates protein synthesis through the phosphorylation and inactivation of eIF4EB-P. Further experimentation is required to test whether CYP2S1-mediated changes in the cyclooxygenase pathway [22,23], stimulating PGE2 synthesis, is the pathway linking CYP2S1-mediated metabolism to mTOR signaling and regulation of cell size in bronchial epithelial cells.

## Conclusions

The data presented in this article represent a novel trancriptomic approach to identify the mode of action for alterations in orphan P450 expression. Although, a transcriptomic approach, alone, is not sufficient to identify endogenous substrates, it can provide clues into the biological significance of CYP2S1-mediated metabolism, and/or compensation for these metabolic shifts. Moreover, it can shed light on potentially novel important physiological pathways. Transcriptome findings of CYP2S1 depleted cells are consistent with previously published cellular phenotypes [23]. The results also identified differential expression of genes involved in the mTOR pathway, a master regulator of cell size, which resulted in the identification of a novel, measurable phenotype (specifically, increase in cell size) in CYP2S1-depleted cells. Additionally, transcriptomic analysis was consistent with proposed endogenous lipid substrates as well as compensatory metabolic shifts in CYPs involved in lipid metabolism. Although this approach provides an excellent way of examining mode of action, it fails to identify specific chemical substrates and metabolic products. Conversely, a metabolomic approach identifies the metabolites but does not establish the physiological impact. Ultimately, a multi-omic approach integrating both transcriptomic and metabolomic analysis, may provide a powerful predictive approach to elucidate endogenous substrates as well as biological significance of orphan P450’s.

## Methods

### RNA isolation

As indicated in our previous publication [23] we used the clones with the most significant difference in CYP2S1 expression for RNA-sequencing analysis (i.e. SCRAM#1 and 759#7). BEAS-2B CYP2S1 depleted cells (759 and 984) and scrambled controls (SCRAM) cells were grown in a six well plates to confluence and washed with PBS. RNA was isolated according to the manufactures protocol (Qiagen RNeasy) and eluted in 20ul of RNAse-free water. RNA quality was analyzed using the Bioanalyzer (Agilent 2100 Bioanalyzer with Agilent RNA 6000 Nano reagents) and samples with RNA integrity number (RIN) between 9 and 10 were sent to the National Center for Genomics Research (NCGR, Santa Fe, NM) for Illumina RNA sequencing or stored for qRT-PCR validation. In total, 3 biological replicates from each genotype (759#7, 984#1, and SCRAM#1) were assessed.

### Library preparation and sequencing

Messenger RNA for 759 and SCRAM samples was isolated from total RNA samples with polyA selection, size selected and prepared into sequencing libraries with the TruSeq RNA sample preparation workflow from Illumina (San Diego, CA). Libraries were sequenced on the Illumina HiSeq 2000 platform, generating 141,896,712 1×50 nt single-end reads, averaging 11,824,726 reads per sample. Raw Illumina reads are available at the Sequence Read Archive at NCBI under the accession: SUB278513.

### Read count based expression

For each sample, raw sequence reads were filtered and aligned to the human reference genome (GRCh37) with GSNAP [49]. Subsequent management of the data was performed by Alpheus [50]. Reads that aligned unambiguously to the human reference were binned based on annotated gene coordinates and summated to estimate expression level. Read count based expression estimates were evaluated for transcriptome-wide qualitative differences with JMP Genomics 6.0. Quantitative differential gene expression analysis was performed with the negative binomial test as implemented in the Bioconductor package DESeq [27]. Genes were identified as differentially expressed if they had an adjusted (Benjamini-Hochberg False Discovery Rate (FDR) method for multiple testing correction) p-value of 0.05 or less.

### Quantitative PCR analysis

cDNA was synthesized, using iScript reverse transcription supermix (BioRad, Hercules, CA), from 1 μg total RNA. Quantitative qRT-PCR was conducted using IQ sybr green supermix (BioRad) in accordance with manufacturers instructions, and performed using the BioRad CFX96. Primer efficiencies were calculated using standard curves obtained from plasmid-based amplicons. The housekeeping gene *ACTB* was selected based on consistency between samples, and samples were normalized to its expression. The qRT-PCR primers used as previously described [23].

### Pathway and functional analysis of differentially expressed genes

To gain biological insight and contextualized differentially expressed genes (as described above), we further analyzed genes that were significantly altered with adjusted p-values of either <0.001 or <0.01. These subsets included 1579 and 2766 total genes, respectively. The open source database for annotation, visualization and integrated discovery (DAVID version 6.7) [28,29] was employed to assess biological function of the selected genes. The functional annotation tool was used to cluster genes based on the degree of association. Functional annotation was set at high stringency and restricted to groups with an enrichment score greater than or equal to 1.3. Gene ontology (GO) terms and KEGG pathway were reported. Functional annotation tool is mainly provided the most relevant gene ontology terms (GO terms) associated with the gene list and we reported the mostly enriched GO term, which is GOTERM_BP_FAT. Additionally we looked into highly affected KEGG pathways with the gene list submitted.

### Cell size determination

BEAS-2B cells were grown in 6 well plates to approximately 80% confluence, trypsinized, and cell diameter and volume were quantified using the Millipore scepter automated cell counter. Trypsinized cellular populations were gated between 10-24 μM to ensure that in-tact cells were identified. The final cell size represents the average cell size from a total of six biological replicates from each genotype.

### PCR pathway array

The human mTOR RT profiler PCR pathway array (SABioscience Qiagen, Cat.no.330231 PAHS-098ZD) was used to validate mTOR regulation identified through RNA seq. RNA was isolated and analyzed as indicated above. cDNA was synthesized (Qiagen, RT first strand kit) and the PCR array was performed according to the manufactures protocol (Qiagen, Cat.no. 330231 PAHS-098ZD). PCR was run on the BIORAD CFX96 real-time cycler and data was analyzed using SABiosciences Excel based PCR array data analysis template (http://www.sabiosciences.com/pcrarraydataanalysis.php). The threshold cycle (C_T_) values, obtained through PCR, were exported to the SABiosciences Excel template. The ∆C_T_ was calculated for each gene in relation to house keeping genes and ∆∆C_T_ was calculated relative to the control sample (SCRAM). Based on the ∆∆C_T_ values fold change (2^(-∆∆CT)^) was reported.

## Abbreviations

CYP2S1: Cytochrome P450 2S1; Beas2B: Bronchial epithelial cells; RNA seq: RNA sequencing; shRNA: Short Hairpin RNA; SCRAM: Scramble; CYPs: Cytochrome P450s; AhR: Arylhydrocarbon Receptor; 3-MC: 3-methylchloranthrene; AQ4N: Anthraquinone; atRA: all trans Retinoic Acid; mRNA: messanger RNA; ACTB: β - actin; GO: Gene ontology; KEGG: Kyoto Encyclopedia of Genes and Genomes; CHO: Chinese Hamster Ovary cells; HaCaT: Human keratinocytes; CRABP: Cellular Retinoic Acid Binding Protein; ADH: Alcohol Dehydrogenases; SDR: short-chain dehydrogenase/reductase; FABP: Fatty Acid Binding Protein; RAR: Retinoic Acid Receptor; PPAR: Peroxisome Proliferating Receptor; AA: Arachidonic Acid; PGG2: Prostaglandins G2; PGE2: Prostaglandins E2; PGD2: Prostaglandins D2; PGH2: Prostaglandins H2; EET: Epoxyeicosatrienoic acid; DEPTOR: DEP domain containing mTOR-interacting protein; eIF4EBP1: Eukaryotic Initiation Factor 4E binding protein 1; FDR: False Discovery Rate; qRTPCR: Quantitative Real Time Polymerase Chain Reaction.

## Competing interests

The authors declare that there are no competing interests.

## Authors’ contributions

TWM – performed the RNA isolation, array studies, DAVID analysis and contributed to writing the manuscript. IEL – wrote sections of the manuscript and critically reviewed the manuscript. NPD – performed qualitative and quantitative transcriptome analyses. JM – reviewed manuscript and methodologies of transcript analyses. AMR – designed the study and wrote the manuscript. All authors read and approved the final manuscript.

## Supplementary Material

Additional file 1 **The list of all genes identified in CYP2S1 depleted (759) and SCRAM BEAS-2B cells.** This is based on read count expression. The differentially expressed genes identified if they had an adjusted (Benjamini-Hochberg False Discovery Rate (FDR) method for multiple testing correction) p-value of 0.05 or less. Click here for file

Additional file 2 **Complete GO BP function annotation results of 995 differentially expressed genes.** These genes exhibit a 2-fold change in expressions that were consistently either up or down-regulated between biological replicates (p < 0.01). Click here for file

Additional file 3 **Differential expressed genes in Retinol metabolism.** Differential gene expression is indicated on the KEGG pathway. Grey indicates no significant change in expression. Gold represents very low expression (i.e. base mean average value is <1.5). Shades of red and blue indicate a significant (p < 0.05) increase and decrease in expression in CYP2S1 depleted cells, respectively. All colored genes except grey indicate significant increases in RNA seq, p < 0.05. Click here for file

Additional file 4 **Complete GO BP function annotation results of 4516 differentially expressed genes.** These genes expressions were consistent between biological replicates (p < 0.05). Click here for file

Additional file 5 **Differential expressed genes in Arachidonic Acid metabolism.** Differential gene expression is indicated on the KEGG pathway. Grey indicates no significant change in expression. Gold represents very low expression (i.e. base mean average value is <1.5). Shades of red and blue indicate a significant (p < 0.05) increase and decrease in expression in CYP2S1 depleted cells, respectively. All colored genes except grey indicate significant increases in RNA seq, p < 0.05. Click here for file
