# Supplementary figures and images for: A transcriptomic approach to elucidate the physiological significance of human cytochrome P450 2S1 in bronchial epithelial cells
Source: BMC Genomics. 2013 Nov 26;14:833. doi: 10.1186/1471-2164-14-833 (PMC3884200; doi:10.1186/1471-2164-14-833)

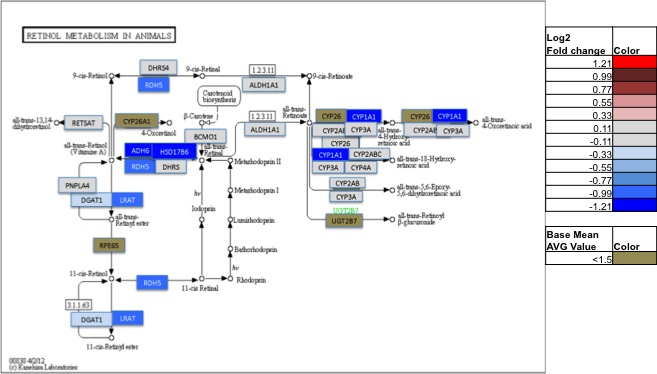

Supplement: Additional file 3 — Differential expressed genes in Retinol metabolism. Differential gene expression is indicated on the KEGG pathway. Grey indicates no significant change in expression. Gold represents very low expression (i.e. base mean average value is <1.5). Shades of red and blue indicate a significant (p < 0.05) increase and decrease in expression in CYP2S1 depleted cells, respectively. All colored genes except grey indicate significant increases in RNA seq, p < 0.05. [file 1471-2164-14-833-S3.jpeg]

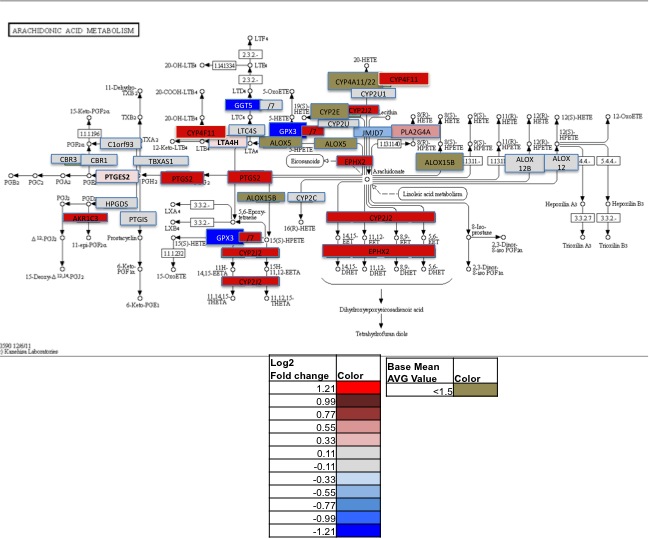

Supplement: Additional file 5 — Differential expressed genes in Arachidonic Acid metabolism. Differential gene expression is indicated on the KEGG pathway. Grey indicates no significant change in expression. Gold represents very low expression (i.e. base mean average value is <1.5). Shades of red and blue indicate a significant (p < 0.05) increase and decrease in expression in CYP2S1 depleted cells, respectively. All colored genes except grey indicate significant increases in RNA seq, p < 0.05. [file 1471-2164-14-833-S5.jpeg]
